# Theoretical Modelling of the Degradation Processes Induced by Freeze–Thaw Cycles on Bond-Slip Laws of Fibres in High-Performance Fibre-Reinforced Concrete

**DOI:** 10.3390/ma15176122

**Published:** 2022-09-03

**Authors:** Rosa Penna, Luciano Feo, Enzo Martinelli, Marco Pepe

**Affiliations:** 1 Department of Civil Engineering, University of Salerno, Via Giovanni Paolo II, 132, 84084 Fisciano, Italy or; 2 TESIS s.r.l., Via Giovanni Paolo II, 132, 84084 Fisciano, Italy

**Keywords:** HPFRC, steel fibres, freezing and thawing durability, meso-scale model, cracked-hinge approach, experimental validation

## Abstract

High-performance fibre-reinforced concrete (HPFRC) is a composite material in which the advantages of fibre-reinforced concrete (FRC) are combined with those of a high-performance concrete (HPC), which mitigates the weaknesses of conventional concrete and improves its overall performance. With the aim to reduce the long-term maintenance costs of structures, such as heavily loaded bridges, HPFRC is highly recommended due to its major durability performance. Specifically, its good antifreezing property makes it suitable for application in cold regions where cyclic freeze–thaw conditions cause the concrete to degrade. In this paper, a numerical simulation of the degradation processes induced by freeze–thaw cycles on bond-slip laws in HPFRC beam specimens has been developed so as to assess their effect on the flexural response of specimens as the fibres’ volume percentage changes. Their cracking strength, postcracking strength, and toughness were predicted, with the present model being able to predict the cracking strength, postcracking strength and toughness of the HPFRC beam element under bending load conditions. Its accuracy was confirmed by comparing the model predictions with experimental results.

## 1. Introduction

Over the last few years, high-performance fibre-reinforced concrete (HPFRC) has been widely used to strengthen ageing concrete structures [1,2,3,4,5,6,7] and to control the crack propagation and displacement in concrete slabs and shells, such as industrial floors, while also improving the seismic response of structural elements, such as columns, beams, and walls [8,9]. Moreover, HPFRC is highly recommended in aggressive environments (e.g., marine environments, higher altitudes, northern areas) due to its high durability which is suitable for long-term structures and heavily loaded bridges to reduce any maintenance costs [10,11]. It is also considered a sustainable material for the manufacturing of small thickness elements without steel rebars, resulting in a reduction in the CO_2_ footprint [12]. It is well-known how HPFRC is a composite material in which the advantages of fibre-reinforced concrete (FRC) are combined with those of a high-performance concrete (HPC), reducing the weaknesses of conventional concrete and improving its durability and mechanical performance. The addition of discontinuous fibres (e.g., steel fibres [13,14,15,16], synthetic fibres [17,18,19,20,21], natural fibres [22,23,24], basalt [25,26]), carbon and glass fibres [27]) in the HPC as well as in the concrete in general is able to significantly reduce its brittle behaviour, thus improving cracking, postcracking strength, and toughness [28,29,30] as well as its durability such as freeze–thaw resistance. Thanks to its dense microstructure, high-performance concrete also has a low permeability, resulting in a good resistance to various external agents such as chloride attacks [31] and carbonation [32] as well as freezing and thawing cycles [33,34,35,36]. Its good antifreezing property makes it suitable for application at both high altitudes and in northern areas where cyclic freeze–thaw conditions are one of the main causes of two types of concrete degradation: surface scaling, which is the loss of cement paste from the exposed surface, and internal crack growth, which makes the concrete crumble and deteriorate. Both phenomena can reduce the quality of concrete throughout its lifetime. Over the last few years, the research on evaluating the freezing resistance of HPFRC has significantly increased, with several relevant achievements having been obtained: in Feo et al. [37], the effects of 75 freeze and thaw cycles on both the dynamic moduli of elasticity, cracking and postcracking strength, as well as the toughness of HPFRC beam specimens reinforced with steel fibres were evaluated; in [38], it was reported how the incorporation of basalt fibres can reduce the influence of freeze–thaw on the damage and failure process of the beam specimen under a bending test; in [39], it was studied how mineral admixtures (e.g., blast furnace slag, fly ash, silica fume, and metakaolin) contained in the HPC matrix possess an excellent frost resistance; in [40], an experimental investigation on the freeze–thaw resistance of HPC containing air-cooled slag (AS) and water-cooled slag (WS) was conducted; in [41], it was explained how adding nanosilica to the concrete makes it durable by enhancing its properties such as impermeability, porosity, and acid resistance. However, based on our knowledge, such studies are experimental and not many predictive models have been proposed that capture the mechanical response of HPFRC, especially under freeze and thaw cycles.

In this study, the previous theoretical model developed by the authors [42], as an extension of a meso-scale formulation of a cracked hinge model implemented in a Matlab code [43], has been improved to predict the effect of freeze and thaw cycles on the flexural behaviour of HPFRC specimens as the fibres’ volume percentage changes. This model is intended to take into account explicitly the behaviour of the two typical “phases” of fibre-reinforced cementitious composites (i.e., the cement-based matrix and the spread reinforcement, as well as with their interaction). The kinematics of the proposed model was inspired by the so-called “cracked-hinge” approach [44,45,46,47,48], but both the random spatial distribution and orientation of fibres and the crack-bridging effect of fibres is explicitly simulated. The present model is able to estimate the cracking, postcracking strength, and toughness of a HPFRC beam element under bending load conditions. Its reliability was confirmed by comparing the model predictions with the experimental results obtained in [37].

## 2. Outline of the Experimental Results

The present study is part of a wider research whose experimental part was already published into details in a previous paper [37]. A brief summary about the obtained results is reported hereafter, for the readers’ sake.

Three different HPFRC mixtures CM0, CM1, and CM2, obtained by fixing the HPC matrix and varying the fibre volume fraction, *Vf*, in the set 0%, 1.25%, and 2.50%, respectively, were examined. Short steel fibres, *Dramix OL 13/0.20* [49], with an aspect ratio, *l_f_/d_f_*, equal to 65 were chosen as the reinforcement of the HPC matrix whose mix design was provided by the manufacturer [50]. For each type of HPFRC mixture, eight standard 150 × 150 × 600 mm prismatic specimens (PS) and five standard 150 × 150 × 150 mm cubic samples (CS) were cast. At the end of the curing period, the prismatic specimens for each HPFRC mixture were subjected to 75 freeze–thaw cycles according to UNI 7087-2017 [51]. 

Subsequently, the prismatic specimens were tested under a four-point bending setup according to UNI 11039-2 [52] in which the vertical load (P) and the corresponding average “Crack Tip Opening Displacement” (*CTOD_avg_*) were monitored during each test. All the cube samples were only tested in compression to evaluate, according to EN 12390-4 [53], the compressive load, *F_u_*, as well as the compressive strength, *f_c_*, for any changes of the fibres’ volume fraction. 

Table 1 reports the average values of the first crack load, *P_lf_*, of the first crack strength, *f*_(*lf,avg*)_, and the equivalent postcracking strengths, *f*_(*eq*(0–0.6),*avg*)_ and *f*_(*eq*(0.6–3),*avg*)_, before and after the freeze–thaw cycles for each type of HPFRC mixture.

Table 2 summarises the average values of the work capacity indices, *U*_(1,*avg*)_ and *U*_(2,*avg*)_, and the ductility indices, *D*_(0,*avg*)_ and *D*_(1,*avg*)_, before and after the freeze–thaw cycles for the two types of HPFRC mixture (CM1 and CM2).

## 3. Theoretical Model

The abovementioned experimental results were here used to improve the *cracked-hinge model* [42] in which a meso-mechanical approach was adopted with the aim of predicting the bending response of HPFRC beam elements under normal environmental conditions.

### 3.1. Assumptions and Formulation

**Figure 1:**
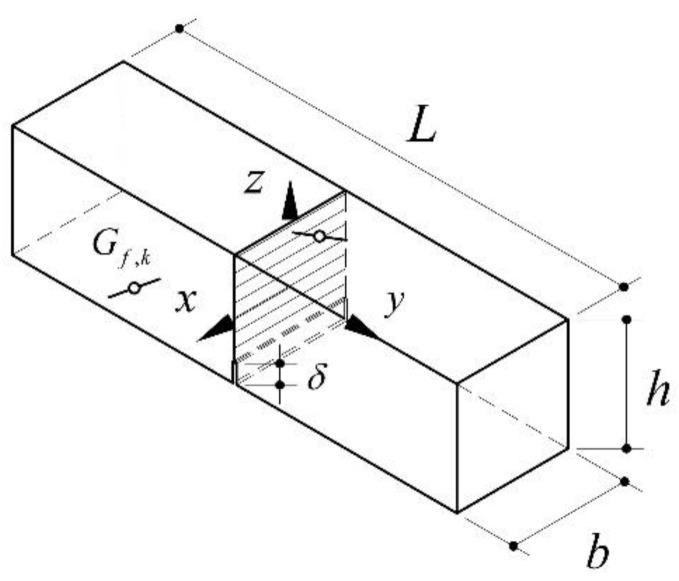
Schematic representation of the 3D HPFRC beam: adapted from [43].

In this new model, however, a different transition zone length and a modified bond-slip law of the fibres were taken into account in order to estimate the effects of freeze–thaw cycles on the flexural behaviour of standard specimens of a length *L*, width *b*, depth *h*, and transversely notched at the midspan section for a depth equal to *h_0_* (Figure 1).

By denoting *x*, *y*, and *z*, the axes of a Cartesian coordinate system originating at the centre of the midspan section, a random distribution of the fibres inside the specimen prismatic volume was generated (by utilising the standard random number generator of Matlab) in which *x*_(*f,k*)_, *y*_(*f,k*)_, and *z*_(*f,k*)_ (with 𝑘 between 1 and *n_f_*), and *α*_(*y,k*)_ and *α*_(*z,k*)_ are the three coordinates of the fibre centroid (*G*_(*f,k*)_) and the two relevant angles, respectively. In order to consider the bridging effect offered by the fibres, the total number of fibres in the midspan section, *n_f_*, was determined as a ratio between the fibres’ volume fraction, *V_f_*, and the cement matrix volume, *V_c_*.

**Figure 2:**
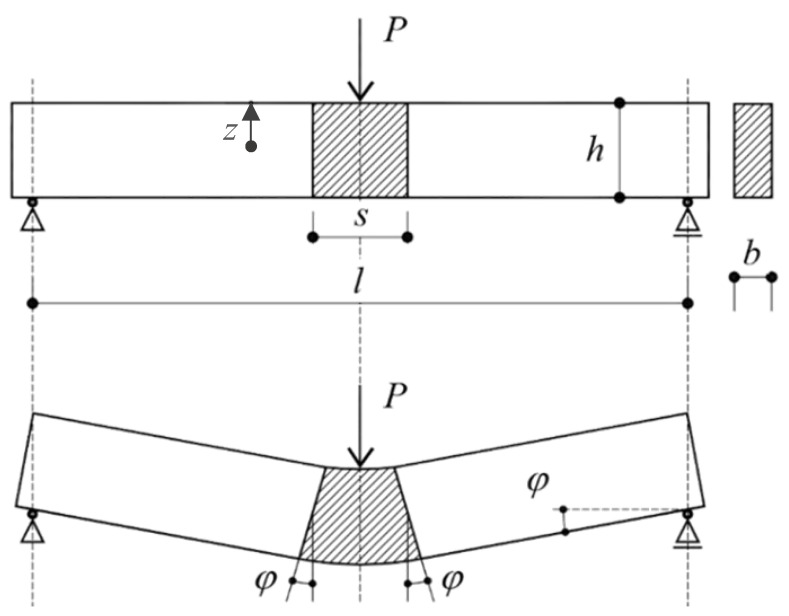
Kinematics of the cracked-hinge model: adapted from [43].

**Figure 3:**
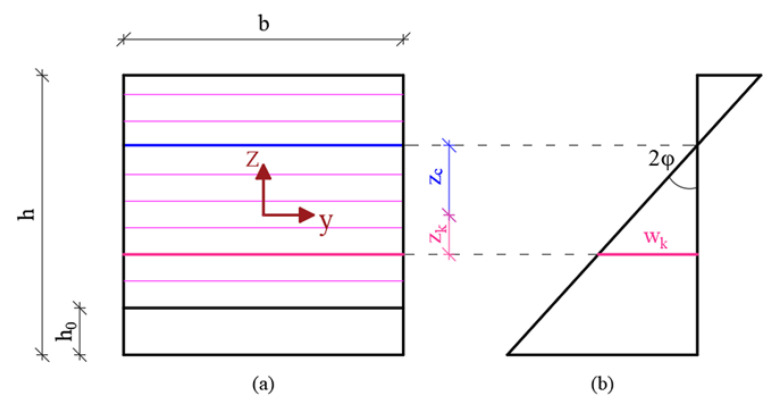
Notched cross-section (**a**) and diagram of normal strain (**b**).

Furthermore, the model was based on the following assumptions:

(i) The flexibility was distributed in the central part of the specimen for a length equal to “s” while a rigid body behaviour was exhibited by the remaining end parts (Figure 2).
(ii) The midspan cross-section was discretized in $n_{c}$ layers as shown in Figure 3. The average axial strain of the *k*-th layer, $\varepsilon_{k}$, before crack formation, and the crack-opening displacement, $w_{k}$, after the crack formation, can be easily expressed for the *k*-th layer (*k =* 1, …, $n_{c}$) as in Equations (1) and (2).

(1) $$\varepsilon_{k}=\frac{2\;\varphi_{j}\cdot \left(z_{c}-z_{k}\right)}{s}$$

(2) $$w_{k}=2\;\varphi_{j}\cdot \left(z_{c}-z_{k}\right)$$

**Figure 4:**
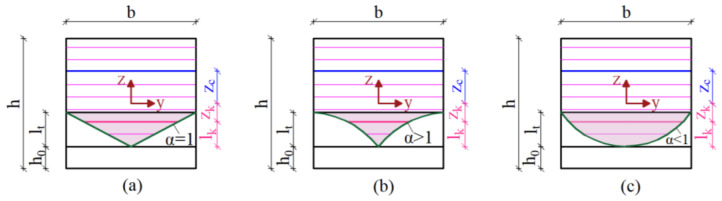
Notched cross-section with exponential law of the reduced width, $b_{k}$, in the transition zone, $l_{t}$: (**a**) linear expression (α = 1) of $b_{k}$, (**b**) exponential expression with α>1, (**c**) exponential expression with α<1.

Equations (1) and (2) are typical of the “cracked hinge” model family (after their original formulations by Hillerborg et al. [46] and Olesen [47]). Specifically, Equation (2) rests on the assumption that plane sections remain plane and Equation (1) on the assumption of a characteristic length “*s*” which can be defined to convert axial displacements (at the numerator of the right-hand side) in axial strains (at the left-hand side).

(iii) Consequently, the average value of the axial stress, $\sigma_{c,k}$, at *k*-th strip can be determined as a function of the axial deformation, $\varepsilon_{k}$, before cracking, or a function of the crack-opening displacements, $w_{k}$, after cracking. The stress–strain and stress–displacement relationships assumed in this paper are reported in Section 3.2.2.
(iv) A transition length, $l_{t}$, was introduced in the notched cross-section (Figure 4), which starts from the top of the notch to the top of the integral part of the section, in order to consider the possible microdamage phenomena produced by the notching process. The mechanical meaning of this quantity is discussed in details in a previous paper [42] and omitted herein for the sake of brevity. Therefore, a reduced value of the width, $b_{k}$, inside the transition zone was considered which can be evaluated with an exponential law as in Equation (3) where $l_{k}$ and α are the distance of the *k*-th strip from the top of the notch and the coefficient of the exponential law, respectively:

(3) $$b_{k}=b\cdot {\left(\frac{l_{k}}{l_{t}}\right)}^{\alpha }$$

(v) The bridging effect offered by the fibres was taken in to account by introducing the action, $F_{k,j}$, mobilised at the *j*-th step of the incremental analysis as in Equation (4):

(4) $$F_{k}\left(z_{f,k};z_{c,j};\;\varphi_{j}\right)=A_{f}\cdot \sigma_{f,k}\left(z_{f,k};z_{c,j};\;\varphi_{j}\right)$$

in which $z_{f,k}$, $z_{c,j}$ and $\varphi_{j}$ are the position of the *k*-th fiber in the cross section, the position of the neutral axis and the rotation of the two rigid blocks at the *j*-th step of the increment analysis (Figure 2), respectively. In particular, the axial stress of the *k*-th fiber, $\sigma_{f,k}$ depends on the sliding of one of the two parts embedded in the two sides of the crack and, therefore, it was correlated to the bond stresses, *τ*, mobilized on its lateral surface due to the crack opening displacement $w_{k}$:

(5) $$\sigma_{f,k}\left(z_{f,k};z_{c,j};\;\varphi_{j}\right)=4\cdot \frac{l_{f}-w_{f,k}\left(z_{f,k};z_{c,j};\;\varphi_{j}\right)\;}{d_{f}}\cdot \tau \left[w_{f,k}\left(z_{f,k};z_{c,j};\;\varphi_{j}\right)\right]$$

Moreover, it should be noted that only a low number of these fibres, $n_{f}^{\prime }<n_{f}$, will cross the crack which will potentially open in the middle of the beam. With the above assumptions, the position *z_c,j_* of the neutral axis for the *j*-th imposed rotation *φ_j_* can be determined by solving the following equilibrium equation along the longitudinal axis which can be written as follows:

(6) $$∆z\cdot b\cdot \left[\overset{n_{t}}{\underset{k=1}{\sum }}{\left(\frac{l_{k}}{l_{t}}\right)}^{\alpha }\cdot \sigma_{c,k}\left(z_{k};z_{c,j};\varphi_{j}\right)+\overset{n_{c}}{\underset{k=n_{t}+1}{\sum }}\sigma_{c,k}\left(z_{k};z_{c,j};\varphi_{j}\right)\right]+\overset{n_{f}^{\prime }}{\underset{k=1}{\sum }}F_{k}\left(z_{f,k};z_{c,j};\varphi_{j}\right)=0$$

where *n_c_* is the number of layers into which the midspan section is discretized (Figure 3) and *n_t_* is the number of layers of reduced width [42]. 

The solution of Equation (6) brings us to determining $z_{c,j}$, at the *j*-th step of the incremental analysis. It is worth highlighting that this solution can only be obtained numerically: the well-known bisection method was employed to determine the actual value of $z_{c,j}$ which should obviously be included in the range between 0 and h–h_0_ (Figure 3).

Then, the corresponding values of the bending moment *M_j_* can be obtained as follows:

(7) $$\begin{array}{cc} M_{j} & =∆z\cdot b\cdot \left[\overset{n_{t}}{\underset{k=1}{\sum }}{\left(\frac{l_{k}}{l_{t}}\right)}^{\alpha }\cdot \sigma_{c,k}\left(z_{k};z_{c,j};\varphi_{j}\right)\cdot \left(\frac{h}{2}-z_{k}\right)+\overset{n_{c}}{\underset{k=n_{t}+1}{\sum }}\sigma_{c,k}\left(z_{k};z_{c,j};\varphi_{j}\right)\cdot \left(\frac{h}{2}-z_{k}\right)\right]\\ & +\overset{n_{f}^{\prime }}{\underset{k=1}{\sum }}F_{k}\left(z_{f,k};z_{c,j};\varphi_{j}\right)\cdot \left(\frac{h}{2}-z_{f,k}\right) \end{array}$$

which is obviously related to the applied vertical load P (in 3-point bending):

(8) $$P_{j}=4\cdot \frac{M_{j}}{l}\;.$$

The corresponding *CTOD_j_* value of can be obtained from Equation (2) by just replacing the generic value of *z_k_* with the position of the crack tip (*z_k_* = −*h*/2 + *h*_0_ from Figue 2).

Therefore, for each value of the imposed rotation *φ_j_*, a couple (*CTOD_j_*, *P_j_*) can be determined, and then, the Force-CTOD graph can be incrementally determined up to failure.

Furthermore, the constitutive laws adopted for the HPC matrix and short steel fibres have the same shape as well as mathematical expressions as those already presented by the authors [42]. However, the unknown parameters in the constitutive laws were calibrated in Section 4 on the experimental results already summarized in Section 2.

### 3.2. Constitutive Laws Assumed in the Present Study

#### 3.2.1. Stress–Strain Relationships for Concrete in Compression and in Tension

**Figure 5:**
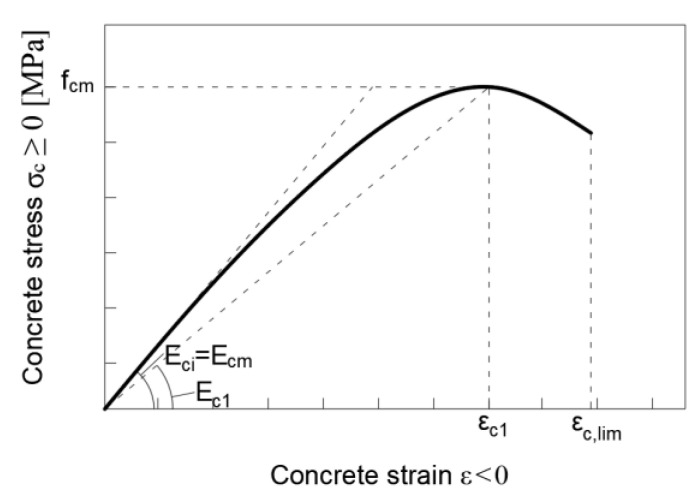
Schematic representation of the stress–strain relation for uniaxial compression.

The stress–strain relationship when the concrete is in compression was described by Equation (7), according to [54], in which η can be calculated as the ratio between the strain, $\varepsilon_{c}$, and the strain at maximum compressive stress, $\varepsilon_{c1}$, can be evaluated as in Equation (8); whereas k is the plasticity number which depends on the elastic modulus in compression, $E_{c}$, and on the secant modulus from the origin to the peak compressive stress, $E_{c1}$, as in Equation (9). The last one can be evaluated as a function of $\varepsilon_{c1}$ and the concrete compressive strength ($f_{cm}$). The schematic representation of the above stress–strain relationship in compression is shown in Figure 5.

(9) $$\sigma_{c}=f_{cm}\cdot \frac{k\cdot \eta -\eta^{2}}{1+\left(k-2\right)\cdot \eta }$$

(10) $$\varepsilon_{c1}=1.60\cdot {\left(\frac{f_{cm}}{10}\right)}^{0.25}$$

(11) $$k=\frac{E_{c}}{E_{c1}}=\frac{E_{c}}{\left(\frac{f_{cm}}{\varepsilon_{c1}}\right)}$$

**Figure 6:**
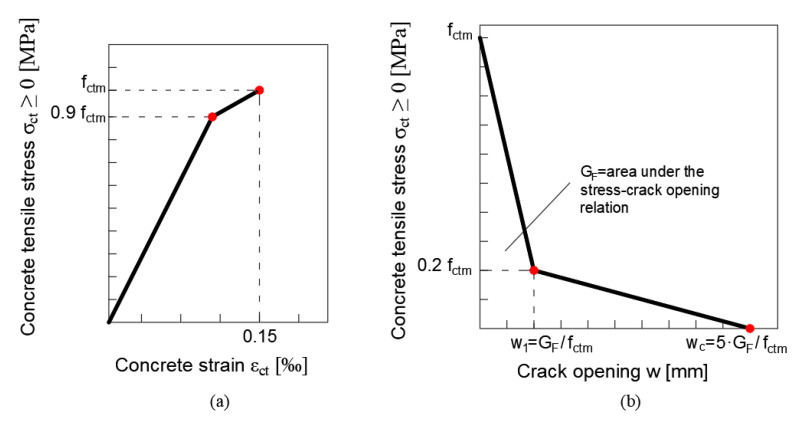
Schematic graph of the stress–strain relation for uniaxial tension: (**a**) for $\varepsilon_{k}\leq 0.00015$, the stress–strain behaviour is described by a bilinear relation; (**b**) for $\varepsilon_{k}>0.00015$, the stress–strain behaviour is described by a softening constitutive stress–crack opening law.

The constitutive law when the concrete is in tension, evaluated according to the “*fictitious crack method*” employed in [44], presents a bilinear relation until the tensile strain of the *k*-th strip, $\varepsilon_{k}$, reaches the conventional value equal to 0.00015 (Figure 6a). A linear elastic behaviour is described by Equation (12a) until the section is uncracked after which the behaviour is expressed by the linear Equation (12b) as follows:

(12) $$\sigma_{ct}=\{\begin{array}{c} E_{ct}\cdot \varepsilon_{ct}\;\;\;\;\;\;\;\;\;\;\;\;\;\;\;\;\;\;\;\;\;\;\;\;\;\;\;\;\;\;\;\;\;\;\;\;\;\;\;\;\;\;\;\;\;\;\;for\;\varepsilon_{ct}\leq 0.9\cdot \frac{f_{ctm}}{E_{ct}}\;\;\;\;\;\;\;\;\;\;\;\;\;\;\;\;\;\;\;\;\;\left(a\right)\\ f_{ctm}\cdot \left(1-0.1\cdot \frac{0.00015-\varepsilon_{ct}}{0.00015-0.9\cdot \frac{f_{ctm}}{E_{ct}}}\right)\;\;\;for\;0.9\cdot \frac{f_{ctm}}{E_{ct}}\leq \varepsilon_{ct}\leq 0.00015\;\;\;\;\left(b\right) \end{array}$$

in which $\sigma_{ct}$, $E_{ct}$, and $\varepsilon_{ct}$ represent the tension stress (in MPa), the elastic modulus under tension load (in MPa), and the tensile strain, respectively, whilst $f_{ctm}$ (in MPa) indicates the tensile strength. Beyond this level, a softening constitutive stress-crack opening law was considered due to the opening of a crack in the *k*-th strip of the cross-section, Equation (13). Consequentially, the residual tension $\sigma_{ct}$ must be expressed as a function of the crack-opening displacement, w, (Figure 6b) in which $w_{1}$ and $w_{c}$ are dependent on the fracture energy as defined in [54]:

(13) $$\sigma_{ct}=\{\begin{array}{c} f_{ctm}\cdot \left(1.0-0.8\cdot \frac{w}{w_{1}}\right)\;\;\;\;\;\;\;\;\;\;\;for\;w\leq w_{1}\;\;\;\;\;\;\;\;\;\;\;\;\;\;\;\;\;\;\;\;\;\;\;\;\;\;\;\;\;\;\;\;\;\;\;\;\;\;\;\;\left(a\right)\\ f_{ctm}\cdot \left(0.25-0.05\frac{w}{w_{1}}\right)\;\;\;\;\;\;\;\;for\;w_{1}\leq w\leq w_{c}\;\;\;\;\;\;\;\;\;\;\;\;\;\;\;\;\;\;\;\;\;\;\;\;\;\;\;\;\;\;\;\left(b\right) \end{array}$$

#### 3.2.2. Modified Bond-Slip Model for Short Steel Fibres

The mathematical relation of the local τ−s constitutive law adopted in this study is provided in Equation (14) in which s was considered equal to the crack-opening displacement $w_{f,k}$ at the level of the *k*-th fibre while the six unknown parameters (i.e., $s_{el},\;s_{R},\;s_{u},\;\tau_{el},\;\tau_{R},\;\tau_{u}$) have to be calibrated using the experimental data of Section 2:

(14) $$\tau =\{\begin{array}{c} \tau_{el}\cdot \frac{s}{s_{el}}\;\;\;\;\;\;\;\;\;\;\;\;\;\;\;\;\;\;\;\;\;\;\;\;\;\;\;\;\;\;\;\;for\;s\leq s_{el}\;\;\;\;\;\;\;\;\;\;\;\;\;\;\;\;\;\;\left(a\right)\\ \tau_{el}+(\tau_{R}-\tau_{el})\frac{s-s_{el}}{s_{R}-s_{el}}\;\;\;\;\;\;for\;s_{el}\leq s\leq s_{R}\;\;\;\;\;\;\;\;\;\left(b\right)\\ \tau_{R}\;\;\;\;\;\;\;\;\;\;\;\;\;\;\;\;\;\;\;\;\;\;\;\;\;\;\;\;\;\;\;\;\;\;\;\;\;\;\;\;for\;s_{R}\leq s\leq s_{u}\;\;\;\;\;\;\;\;\;\left(c\right) \end{array}$$

Consequently, the nonlinear bond-slip law is divided in three different branches, as expressed in Equation (12):

- a linear-elastic behaviour up to the stress level corresponding to matrix tensile strength, identified by the two parameters $s_{el}$, $\tau_{el}$;
- a hardening behaviour, characterized by the formation of many microcracks in the HPFRC mix, identified by the two parameters $s_{R}$ and $\tau_{R}$;
- a constant behaviour defined by the two parameters $s_{u}$ and $\tau_{u}$.

The curve presents an adequate “shape” for describing the global pull-out response of short steel fibres embedded within the HPC matrices. For the sake of simplicity, the current assumption includes the effect of the fibre orientation in space (from 0° to 45°) with respect to the matrix surfaces, as demonstrated in a previous study [55]. However, more accurate assumptions could be formulated with the aim to take into account both the axial deformation of fibres (which is neglected in this study, as it is focused on “short” fibres) and the aforementioned effect of fibre orientation with respect to the transverse section of the specimen at midspan.

## 4. Inverse Identification of the Relevant Material Laws

An inverse identification procedure was carried out with the aim to determine the values of the model parameters that lead to minimizing the difference between the measured and predicted Force-CTOD curves. Specifically, the cylindrical compression strength, $f_{cm}$, the transition length $l_{t}$, the exponential parameter, α, and the six parameters of the bond-slip law (i.e., $s_{el},\;s_{R},\;s_{u},\;\tau_{el},\;\tau_{R},\;\tau_{u}$) were considered as variable with some quantitative restrictions (e.g., *s*_*el*_ < *s*_*R*_ < *s*_*u*_, *τ*_*el*_ < *τ*_*R*_ and *τ*_*R*_ = *τ*_*u*_) intended at respecting the mechanical consistency of the model. In order to evaluate how the values of these parameters depend on the effect of freeze–thaw cycles, several numerical simulations were carried out. In particular, three groups of 100 simulations each, assuming $n_{c}=50$ and s = 300 mm, were run as described below.

**Figure 7:**
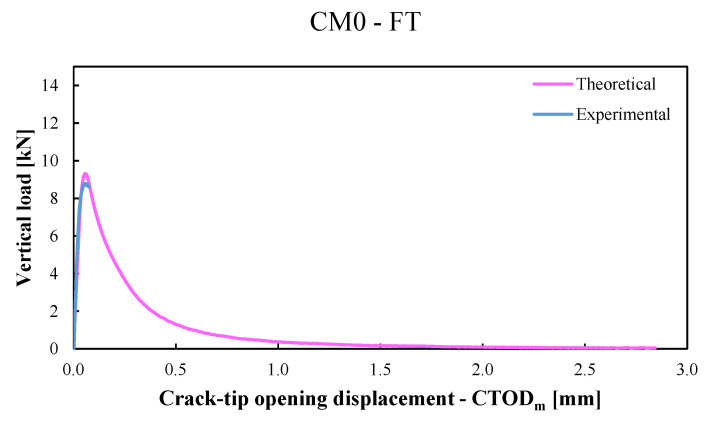
The average experimental $P-CTOD_{,avg}$ curve (light-blue line) versus the average numerical $P-CTOD_{,avg}$ curve (pink line) for conditioned CM0 specimens (labelled CM0-FT) obtained with the present model.

- In the first one, the cylindrical compression strength, $f_{cm}$, the transition length $l_{t}$, and the exponential parameter, α, were calibrated on the flexural response of the conditioned CM0 specimens (labelled CM0-FT). Experimentally, a 21% reduction in the cylindrical compression strength, $f_{cm}$, was observed on the conditioned specimens compared to not conditioned ones. This reduction was taken in account to calibrate the value of the transition length, $l_{t}$, whose value, in the present model, was assumed equal to 85 mm (with an increase of 21% compared to that used in the previous model [42] in which the flexural behaviour of unconditioned CM0 specimens (labeled CM0-NFT) was predicted with a transition length, $l_{t}$, equal to 70 mm). In both models, the coefficient of the exponential law, α, was considered constant and equal to 0.40 (Table 3). Figure 7 shows both the average experimental $P-CTOD_{,avg}$ curve (light-blue line) and the average numerical $P-CTOD_{,avg}$ curve (pink line) obtained with the present model employed for the CM0-FT specimens.
- In the second one, the six parameters of the bond-slip law (i.e., $s_{el},\;s_{R},\;s_{u},\;\tau_{el},\;\tau_{R},\;\tau_{u}$) were calibrated on the flexural response of conditioned CM1 specimens (labelled CM1-FT). A 13% reduction in the parameter $\tau_{el}$ was adopted in the calibration of the conditioned specimens compared to the unconditioned ones.
- In the last one, only the parameter, $\tau_{el}$, was calibrated again on the flexural response of conditioned CM2 specimens (labelled CM2-FT) while all the other parameters were considered constant. A 19% reduction in the parameter $\tau_{el}$ was adopted for the conditioned specimens compared to the unconditioned ones. Moreover, as in [42], a 20% reduction in the fibres’ volume fraction, $V_{f}$, was considered in order to take into account the nonuniform fibre distribution.

**Figure 8:**
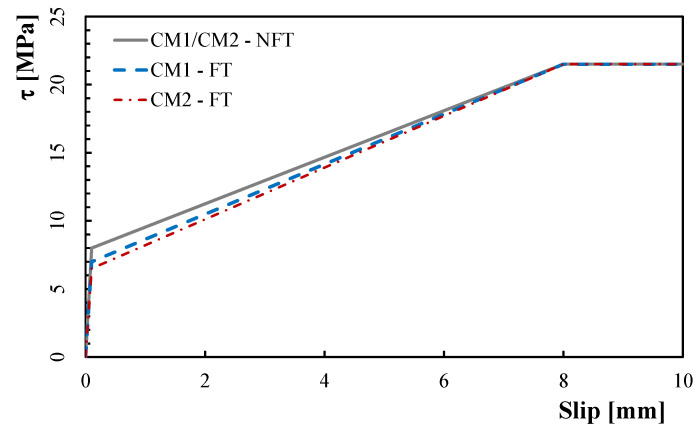
Calibration of the local bond-slip law for unconditioned and conditioned specimens.

**Figure 9:**
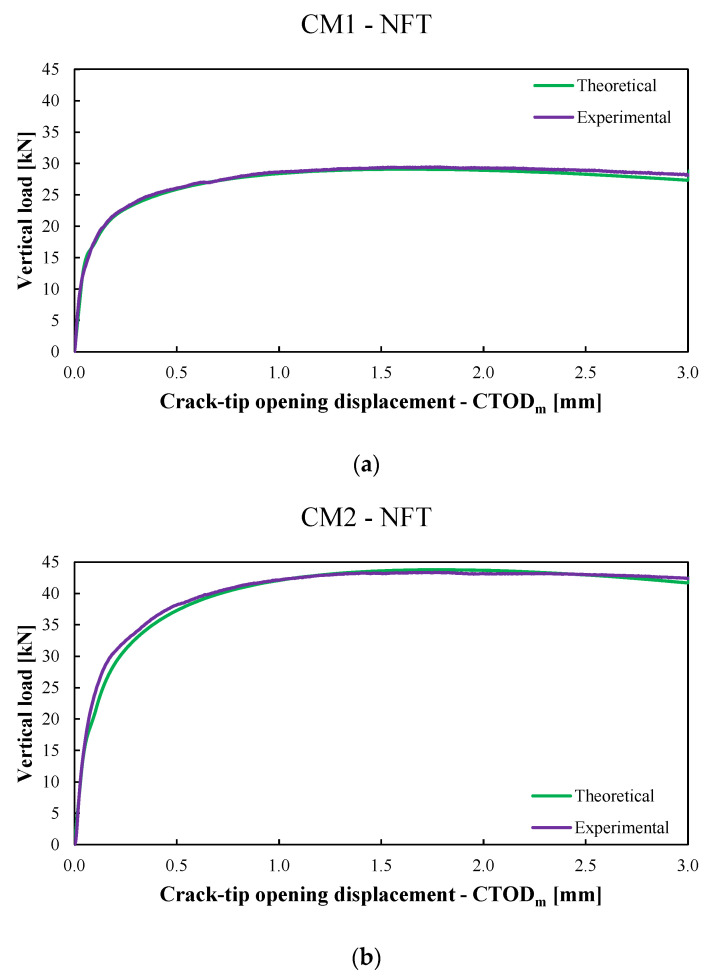
(**a**). The average experimental $P-CTOD_{,avg}$ curve (violet line) versus the average numerical $P-CTOD_{,avg}\;$ curve (green line) for unconditioned CM1 specimens (labelled CM1-NFT) obtained with the previous model of Ref. [42]. (**b**). The average experimental $P-CTOD_{,avg}\;$ curve (violet line) versus the average numerical $P-CTOD_{,avg}\;$ curve (green line) for unconditioned CM2 specimens (labelled CM2-NFT) obtained with the previous model of Ref. [42].

**Figure 10:**
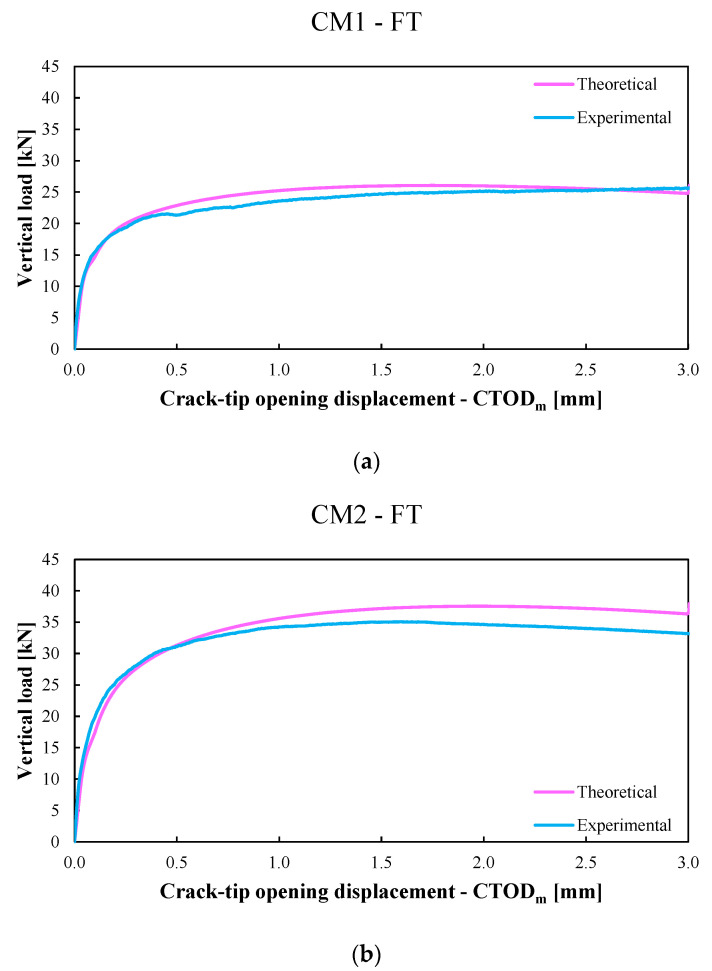
(**a**). The average experimental $P-CTOD_{,avg}$ curve (light-blue line) versus the average numerical $P-CTOD_{,avg}\;$ curve (pink line) for conditioned CM1 specimens (labelled CM1-FT) obtained with the present model. (**b**). The average experimental $P-CTOD_{,avg}\;$ curve (light-blue line) versus the average numerical $P-CTOD_{,avg}\;$ curve (pink line) for conditioned CM2 specimens (labelled CM2-FT) obtained with the present model.

The results of the last two calibrations were listed in Figure 8 and Table 4, while Figure 9a and Figure 10a show the comparison between the average experimental $P-CTOD_{,avg}$ curve (violet line) and the average numerical $P-CTOD_{,avg}$ curve (green line) obtained with the previous model for the CM1-NFT and CM2-NFT specimens, respectively. Whereas Figure 9b and Figure 10b show the comparison between the average experimental $P-CTOD_{,avg}$ curve (light-blue line) and the average numerical $P-CTOD_{,avg}$ curve (pink line) used with the present model for the CM1-FT and CM2-FT specimens, respectively.

## 5. Results

The model developed by the authors [42] was, here, used to predict the postcracking response of HPFRC beam elements under freeze–thaw cycles. 

In order to assess the model accuracy, the theoretical and experimental results of the CM1 and CM2 specimens were compared: first, the average values of the two equivalent postcracking strengths after the freeze–thaw cycles, $f_{eq\left(0-0.6\right),avg}^{FT}$ and $f_{eq\left(0.6-3\right),avg}^{FT}$, are listed in Table 5, for the CM1 and CM2 mixtures, in which the values were compared with those obtained before the freeze–thaw cycles of Ref. [42], $f_{eq\left(0-0.6\right),avg}^{NFT}$ and $f_{eq\left(0.6-3\right),avg}^{NFT}$; second, the average values of the two working capacity indices after the freeze–thaw cycles, $U_{1,avg}^{FT}$ and $U_{2,avg}^{FT}$, are summarised in Table 6, for the CM1 and CM2 mixtures, in which the values were compared with those obtained before the freeze–thaw cycles of Ref. [42], $U_{1,avg}^{NFT}$ and $U_{2,avg}^{NFT}$.

**Figure 11:**
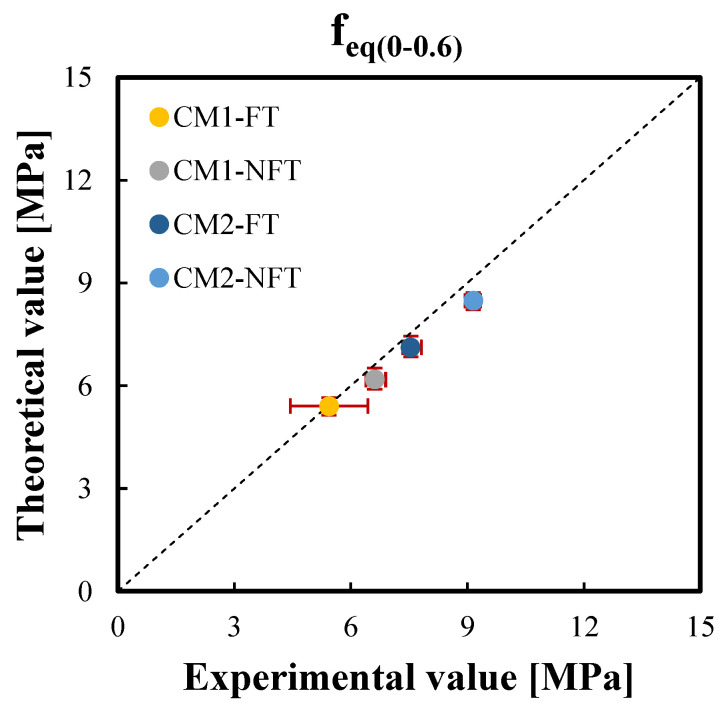
Correlation between the experimental and theoretical average values of the equivalent postcracking strengths after the freeze–thaw cycles, $f_{eq\left(0–0.6\right),avg}^{FT}$ and before the freeze–thaw cycles of Ref. [42], $f_{eq\left(0–0.6\right),avg}^{NFT}$ for the CM1 and CM2 mixtures.

**Figure 12:**
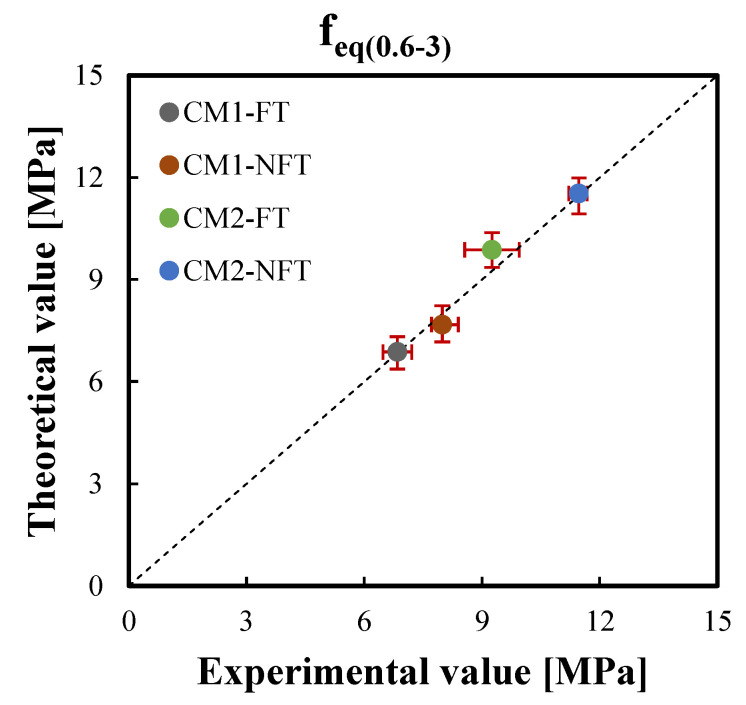
Correlation between the experimental and theoretical average values of the equivalent postcracking strengths after the freeze–thaw cycles, $f_{eq\left(0.6–3\right),avg}^{FT}$, and before the freeze–thaw cycles of Ref. [42], $f_{eq\left(0.6–3\right),avg}^{NFT}$, for the CM1 and CM2 mixtures.

**Figure 13:**
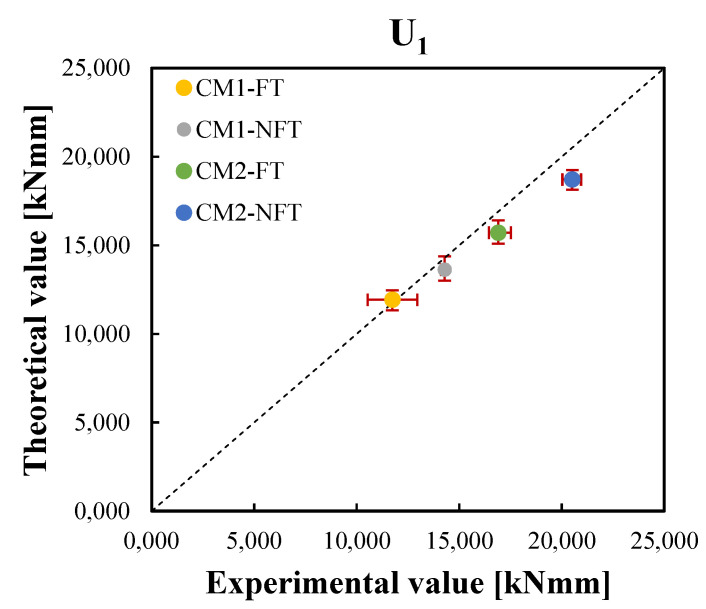
Correlation between the experimental and theoretical average values of the working capacity indices after the freeze–thaw cycles, $U_{1,avg}^{FT}$, and before the freeze–thaw cycles of Ref. [42], $U_{1,avg}^{NFT}$, for the CM1 and CM2 mixtures.

**Figure 14:**
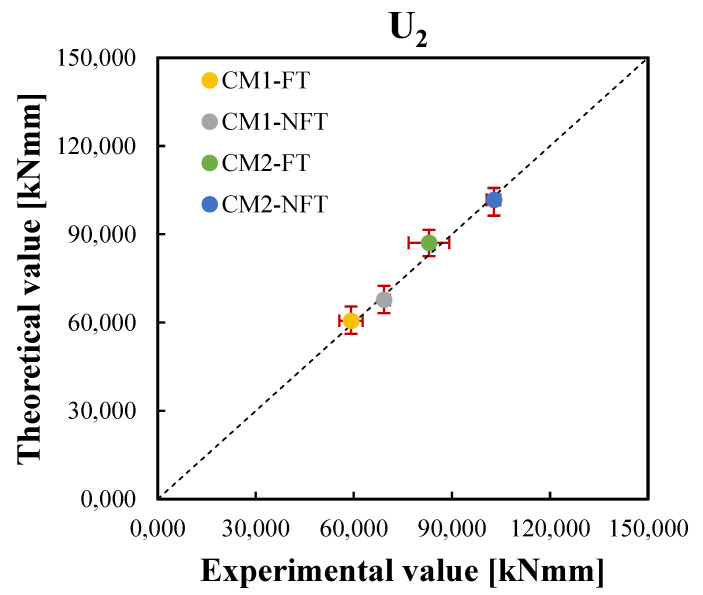
Correlation between the experimental and theoretical average values of the working capacity indices after the freeze–thaw cycles, $U_{2,avg}^{FT}$, and those before the freeze–thaw cycles of Ref. [42], $U_{2,avg}^{NFT}$, for the CM1 and CM2 mixtures.

The correlation graphs of the two equivalent postcracking strengths were plotted in Figure 11 and Figure 12, respectively, while Figure 13 and Figure 14 show the correlation graphs of the average values of the two working capacity indices $U_{1}$ and $U_{2}$, respectively. For each point, the red bars represent the standard deviation of the experimental and predicted values. It was noted how the convergence with the present model was good so that a lower standard deviation was observed.

## 6. Conclusions

The present paper was aimed at scrutinizing the effects of freeze–thaw cycles on the fundamental mechanical behaviour of the “components” controlling the structural behaviour of HPFRC members in bending. Specifically, based on a series of experimental results obtained by the first two authors in a previous study [37], a simplified “cracked-hinge” model was considered with the aim to identify both the concrete constitutive relationship and the bond-slip law of fibres in “unconditioned” and “conditioned specimens”.

Based on the results obtained from the aforementioned inverse identification procedure, the following main considerations can be drawn out:

- The freeze–thaw cycles effect the cylindrical compression strength, $f_{cm}$, the transition length $l_{t}$, and the bond-slip law of fibres, which confirms their significance as relevant parameters controlling the resulting response of HPFRC specimens;
- Table 3 shows that the compressive strength *f_cm_* undergoes a substantial reduction (in the order of 20%) as a result of the degradation processes induced by the FT cycles;
- as for the transition zone, which is a peculiar aspect of the considered model, a moderate increase in its the depth (from 70 mm to 85 mm) can be identified after the FT cycles, whereas its shape (controlled by the exponent α) does not change;
- Table 4 points out that the bond-slip law of fibres is also affected by the FT cycles, as, specifically, the elastic limit stress, *τ_el_*, (and, consequently, the initial elastic stiffness of the same law) reduces by about 15%, with no changes in the other parameters;
- however, under the designers’ standpoint (and besides the specific values obtained in the present study), it should be noted that this change affects both serviceability and ultimate limit states in the structural response.

Finally, the generally good agreement between the experimental data and the values obtained from the identified model confirms the mechanical consistency of the latter and its potential accuracy. However, further experimental results are needed to calibrate general relationships between the main parameters controlling the bond-slip law of fibres and the actual number of freeze–thaw cycles: this will be part of the future developments of the present research.

## Figures and Tables

**Table 1:** The average values of the first crack load, *P_lf_*, of the first crack strengths, *f*_(*If,avg*)_, and of the equivalent postcracking strengths, *f*_(*eq*(0–0.6),*avg*)_ and *f*_(*eq*(0.6–3),*avg*)_, before (NFT) and after the freeze–thaw cycles (FT) for each type of HPFRC mixture (CM0, CM1, and CM2).

Mix.	$P_{If,avg}^{NFT}$	$P_{If,avg}^{FT}$	$f_{If,avg}^{NFT}$	$f_{If,avg}^{FT}$	$f_{eq\left(0–0.6\right),avg}^{NFT}$	$f_{eq\left(0-0.6\right),avg}^{FT}$	$f_{eq\left(0.6–3\right),avg}^{NFT}$	$f_{eq\left(0.6–3\right),avg}^{FT}$
	[kN]	[kN]	[MPa]	[MPa]	[MPa]	[MPa]	[MPa]	[MPa]
CM0	11.213	9.105	3.05	2.477	-	-	-	-
CM1	14.489	12.538	4.013	3.475	6.617	5.435	7.99	6.845
CM2	18.595	16.175	5.06	4.327	9.15	7.537	11.473	9.255

**Table 2:** The average values of the work capacity indices, *U*_(1,*avg*)_ and *U*_(2,*avg*)_, and ductility indices, *D*_(0,*avg*)_ and *D*_(1,*avg*)_, before (NFT) and after the freeze–thaw cycles (FT) for the two types of HPFRC mixture (CM1 and CM2).

Mix.	$U_{1,avg\;}^{NFT}$	$U_{1,avg\;}^{FT}$	$U_{2,avg\;}^{NFT}$	$U_{2,avg\;}^{FT}$	$D_{0,avg\;}^{NFT}$	$D_{0,avg\;}^{FT}$	$D_{1,avg\;}^{NFT}$	$D_{1,avg\;}^{FT}$
	[kNmm]	[kNmm]	[kNmm]	[kNmm]	[-]	[-]	[-]	[-]
CM1	14,283.43	11,742.40	69,226.73	59,175.15	1.647	1.565	1.265	1.265
CM2	20,508.47	16,895.83	102,877.93	82,992.00	1.837	1.747	1.257	1.250

**Table 3:** Calibration of the input data for unconditioned CM0 specimens (labelled CM0-NFT) and for conditioned CM0 specimens (labelled CM0-FT) used in the previous model of Ref. [42] and in the last one, respectively.

Specimen Designation	$f_{cm}$	$l_{t}$	α	Model
	[MPa]	[mm]	[-]	
CM0-NFT	53.0	70.0	0.4	Ref. [42]
CM0-FT	42.0	85.0	0.4	Present paper

**Table 4:** Calibration of the six parameters in the local bond-slip law for unconditioned CM1 and CM2 specimens (labelled, respectively, CM1-NFT and CM2-NFT) as well as for conditioned CM1 and CM2 specimens (labelled, respectively, CM1-FT and CM2-FT) used in the previous model of Ref. [42] and in the last one, respectively.

Series	$s_{el}$	$s_{R}$	$s_{u}$	$\tau_{el}$	$\tau_{R}$	$\tau_{u}$	Model
	[mm]	[mm]	[mm]	[MPa]	[MPa]	[MPa]	
CM1-NFT	0.10	8.00	10.00	8.00	21.50	21.50	Ref. [42]
CM1-FT	0.10	8.00	10.00	7.00	21.50	21.50	Present paper
CM2-NFT	0.10	8.00	10.00	8.00	21.50	21.50	Ref. [42]
CM2-FT	0.10	8.00	10.00	6.50	21.50	21.50	Present paper

**Table 5:** Comparison between the experimental and theoretical average values of the two equivalent postcracking strengths after the freeze–thaw cycles, $f_{eq\left(0–0.6\right),avg}^{FT}$ and $f_{eq\left(0.6–3\right),avg}^{FT}$, and those before the freeze–thaw cycles of Ref. [42], $f_{eq\left(0–0.6\right),avg}^{NFT}\;$ and $f_{eq\left(0.6–3\right),avg}^{NFT}$, for the CM1 and CM2 mixtures.

Results	CM1				CM2			
	$f_{eq\left(0–0.6\right),avg}^{NFT}$	$f_{eq\left(0–0.6\right),avg}^{FT}$	$f_{eq\left(0.6–3\right),avg}^{NFT}$	$f_{eq\left(0.6–3\right),avg}^{FT}$	$f_{eq\left(0–0.6\right),avg}^{NFT}$	$f_{eq\left(0–0.6\right),avg}^{FT}$	$f_{eq\left(0.6–3\right),avg}^{NFT}$	$f_{eq\left(0.6–3\right),avg}^{FT}$
	[MPa]	[MPa]	[MPa]	[MPa]	[MPa]	[MPa]	[MPa]	[MPa]
Experimental	6.617	5.435	7.990	6.845	9.150	7.537	11.473	9.255
Theoretical	6.179	5.411	7.672	6.871	8.486	7.125	11.532	9.871
Percentage difference (%)	6.61	0.45	3.98	0.39	7.26	5.47	0.51	6.66

**Table 6:** Comparison between the experimental and theoretical average values of the two working capacity indices after the freeze–thaw cycles, $U_{1,avg}^{FT}$ and $U_{2,avg}^{FT}$, and those before the freeze–thaw cycles of Ref. [42], $U_{1,avg}^{NFT}$ and $U_{2,avg}^{NFT}$, for the CM1 and CM2 mixtures.

Results	CM1				CM2			
	$U_{1,avg}^{NFT}$	$U_{1,avg}^{FT}$	$U_{2,avg}^{NFT}$	$U_{2,avg}^{FT}$	$U_{1,avg}^{NFT}$	$U_{1,avg}^{FT}$	$U_{2,avg}^{NFT}$	$U_{2,avg}^{FT}$
	[kNmm]	[kNmm]	[kNmm]	[kNmm]	[kNmm]	[kNmm]	[kNmm]	[kNmm]
Experimental	14,283.43	11,742.40	69,226.73	59,175.15	20,508.47	16,895.83	102,877.93	82,992.00
Theoretical	13,625.51	11,930.62	67,667.93	60,606.61	18,711.24	15,709.63	101,710.39	87,061.56
Percentage difference (%)	4.61	1.60	2.25	2.42	8.76	7.02	1.13	4.90

## Data Availability

Not applicable.
